# HbA1c – A predictor of dyslipidemia in type 2 Diabetes Mellitus

**DOI:** 10.12669/pjms.36.6.2000

**Published:** 2020

**Authors:** Saera Suhail Kidwai, Ayesha Nageen, Farhat Bashir, Jamal Ara

**Affiliations:** 1 Prof. Saera Suhail Kidwai, MCPS, FCPS (Internal Medicine), Department of Medicine, United Medical and Dental College, Karachi, Pakistan; 2 Ayesha Nageen, FCPS (Internal Medicine). Assistant Professor, Department of Medicine, United Medical and Dental College, Karachi, Pakistan; 3 Prof. Farhat Bashir, MCPS, FCPS (Internal Medicine). Department of Medicine, United Medical and Dental College, Karachi, Pakistan; 4 Jamal Ara, FCPS. (Internal Medicine), M.Phil (UK). Head of Medicine Department, Department of Medicine, United Medical and Dental College, Karachi, Pakistan

**Keywords:** Type 2 Diabetes mellitus, Dyslipidemia, HbA1c, Lipid Profile

## Abstract

**Objective:**

This study was aimed to determine the predictive value of HbA1c in detecting dyslipidemia in patients with Type-2 Diabetes Mellitus.

**Methods:**

A total of 142 consecutive patients of Type-2 diabetes mellitus were recruited in this study after informed consent. The study was conducted for 6 months from January 2019 – June 2019 in Creek General Hospital, Korangi, Creek, Karachi. Demographic data and detailed history was taken. A complete systemic examination was done for any complications or co-morbids present and related investigations were performed including Fasting lipid profile (CHO, TG’s, HDL, LDL, CHO/HDL), serum HbA1c, Creatinine and ECG. Data is analyzed on SPSS 16 for mean, frequencies and correlations. Pearsons Chi square test is used for analyses of Correlation

**Results:**

In a total of 142 Type-2 diabetic patients 39(27.5%) were Males and 103(72.5%) were females with a male to female ratio of 1: 2.6. Mean age was 54.9yrs ± 10.7SD. Mean duration of diabetes was 7.37yrs ±5.64 SD years. Mean BMI is 26.8 ± 3.67kg/m2. 27(19.01%) patients had HbA1c ≤ 7% whereas 115(80.9%) had >7%. 81(57.04%) patients had dyslipidemia. HbA1c exhibited direct correlations with BMI, cholesterol, TG’s and LDL and inverse correlation with HDL with significant P value of <.05. TG’s were found significantly higher in females when compared with male patients. In addition, Metabolic syndrome also showed a strong correlation with increasing HbA1c levels especially in female gender (P0.001).

**Conclusion:**

The results of our study indicates that HbA1c can be used not only as a useful biomarker of long-term glycaemic control but also a good predictor of lipid profile.

## INTRODUCTION

Diabetes Mellitus attributes to approximately 5% of deaths each year.^1^ Epidemiological studies have demonstrated that Type-2 diabetes mellitus (DM) is a well-known risk factor for the development of cardiovascular disease (CVD), cerebrovascular accidents (CVA), and peripheral vascular diseases.^2^

Diabetic dyslipidemia which is the major contributor of micro and macro vascular complications is generally characterized by an increased plasma triglyceride (TG), low-density lipoprotein (LDL) and apolipoprotein B concentration as well as a decreased high-density lipoprotein cholesterol (HDL-C) concentrations. Dyslipidemia is a well- recognized and modifiable risk factor that if recognized early can result in instituting aggressive cardiovascular preventive management.^3^

In Type-2 DM, the relative insulin deficiency and decreased adiponectin causes decrease lipoprotein lipase activity resulting in high levels of low-density lipoprotein (LDL), triglyceride and low levels of high-density lipoprotein (HDL). Qualitative defects in LDL are also seen in Type-2 diabetes including atherogenic, glycated or oxidized LDL further amplifying the risk of atherogenesis.^4^,^5^

The diabetes complications and control trial (DCCT) established HbA1c as the gold standard to assess glycemic control.^6^ It is considered to be the gold standard marker and has shown significant relationship with lipid profile of Type-2 diabetic patients in several research studies. To date enough published data is not available from our setting to address a linear or inverse linear relationship that might exist between HbA1c and diabetic dyslipidemia from our region and since the phenotypical and genotypical differences of this region varies from other Asian, European and Western regions, it justifies the objective of this study. We have designed this study to determine if such a positive relationship exists as it would help in early and aggressive therapeutic interventions of the targeted patient thus reducing the morbidity and mortality as well as the heavy cost that has to be tolerated later to meet the demands of managing the cardiovascular, cerebrovascular and other micro or macrovascular complications.

## METHODS

This cross sectional comparative cohort study was conducted in department of Medicine in Creek General Hospital, Korangi, Creek, Karachi. One hundred and fifty patients of diabetes mellitus were randomly selected from the outdoor and indoor patient visiting the Medical Out Patient Department and Diabetic Clinic after taking informed consent. Ethical review boards approval was taken from the ethical review committee of the institution (UMDC/Ethics/2018/26/12/253, Dated: 26th Dec, 2018). Sampling technique is non probability Convenience sampling. The age group selected was 40-60 years irrespective of gender. History was taken, BMI recorded and relevant examination was done.

Patients, who were pregnant or had severe heart failure, renal failure, uncontrolled or refractory hypertension and severe concurrent illness were excluded from the study. Serum samples were analyzed for fasting and random blood sugars, total cholesterol, triglycerides, LDL and HDL cholesterol, urea and creatinine using commercially available kits. Ultrasound abdomen for the size of kidney and 24 hours. urinary protein and creatinine clearance were also taken into consideration when indicated along with ECG to identify the existing chronic kidney disease and ischemic heart disease if present. Glycosylated hemoglobin (HbA1c) was estimated by Boronate affinity assay.

Diabetic patients were classified into two groups as per their glycemic index; first group consists of patients with HbA1c value ≤7.0% representing fair control and second group comprising of patients with HbA1c value >7.0% represents poor control. According to National Cholesterol Education Program Adult Treatment Panel (NCEP ATP III) definition Asian modified, Hypercholesterolemia is defined as total cholesterol > 200 mg/dl, high low -density lipoprotein cholesterol (LDL- C) when value > 100 mg/dl, hypertriglyceridemia as triglyceride > 150 mg/dl and low High-density lipoprotein cholesterol (HDL-C) when value is < 40 mg/dl.

Dyslipidemia is defined by presence of one or more than one abnormal serum lipid concentration from above.^7^ Similarly, the presence of three or more of any of the following are considered as having Metabolic Syndrome:

1. Waist circumference (WC) greater than 90 cm in men and 80 cm in women
2. Serum triglycerides (TG) level of at least 150 mg/dL (1.69 mmol/L)
3. High-density lipoprotein cholesterol (HDL-C) level of less than 40 mg/dL (1.04 mmol/L) in men and 50 mg/dL (1.29 mmol/L) in women
4. Blood pressure of at least 130/85 mm Hg; 5) Serum fasting glucose level of at least 110 mg/dL or 6.05 mmol/L.

All values are expressed as mean ± standard deviation of mean. Statistical analysis was done by independent samples t- test. The results were taken significant if p<0.05. The levels of serum lipid profile i.e. serum Cholesterol, LDL, Triglycerides, HDL were taken as dependent variable and age, gender, duration of diabetes, BMI, and HbAIc were used as independent variable, Pearson chi square test is applied to find out the correlation. SPSS 20 is used for statistical analyses.

## RESULTS

A total of 142 Type-2 diabetic individuals were included in this study. The mean duration of diabetes in patients was 7.37 ± 5.64 years. Male: Female ratio is 39:103. Mean age is 54.95±10.6. The mean BMI was 26.8 ± 3.67kg/m2 in diabetic patients. The demographics and lipid profile in both genders which shows significant difference in the lipid profile of both genders especially the LDL and TG’s level in females which are above the normal range as compared to males, in addition, male population has a 0.65% higher HbA1c level than female are shown in Table-I. However, CHO/HDL ratio is slightly higher in females.

**Table-I:** Demographics of male and female subjects with BMI, HbA1c and lipid profile.

	Total (n#142)	Male (n# 39)	Female(n#103)
Age	55.13±10.65	56.48±9.02	54.64±11.1
DMYRS	7.25±5.25	7.70±4.98	7.08±5.36
BMI	26.80±3.67	26.40±2.96	26.94±3.9
CHO	177.38±48.5	173.36±57.5	178.83±45
TGs	165.92±59.39	149.73±58.2	171.81±59
LDL	106.10±36.33	99.66±43.4	108.44±33
HDL	39.52±12.8	42.80±15.6	38.33±11
CHO/HDL	4.87±2.58	4.35±1.3	5.06±2.8
HbA1c	9.33±2.28	9.74±2.25	9.19±2.3

BMI, Cholesterole (CHO), Low density lipoproteins (LDL) and Triglycerides (TG’s) are significantly correlated with HbA1c whereas inverse relation is noted with the High-density lipoproteins (HDL). Table-II

**Table-II:** Pearson’s Correlation of HbA1c with age, duration of diabetes, BMI and Lipid profile.

		HbA1c
HbA1c	Pearson Correlation	1
	Sig. (2-tailed)	
	N	142
Age	Pearson Correlation	0.040
	Sig. (2-tailed)	0.633
	N	142
Dmyrs	Pearson Correlation	-0.013
	Sig. (2-tailed)	0.874
	N	142
BMI	Pearson Correlation	0.243(**)
	Sig. (2-tailed)	0.004
	N	142
CHO	Pearson Correlation	0.179(*)
	Sig. (2-tailed)	0.033
	N	142
TGs	Pearson Correlation	0.262(**)
	Sig. (2-tailed)	0.002
	N	142
LDL	Pearson Correlation	0.166(*)
	Sig. (2-tailed)	0.048
	N	142
HDL	Pearson Correlation	-0.218(**)
	Sig. (2-tailed)	0.009
	N	142
CHO/HDL	Pearson Correlation	-0.042
	Sig. (2-tailed)	0.617
	N	142

The comparison of different variables when categorized according to the blood sugar control that is good control when HbA1c ≤ 7 % and poor control when it is > 7%, it shows BMI, CHO, LDL and TG’s again are significantly high in uncontrolled diabetes with mean HbA1c of 10.23±1.99. Table-III.

**Table-III:** Demographics and lipid profile of subjects when categorized in HbA1c ≤ 7% (fair control) and >7% (poor control).

HBA1c_grp				

	up to 7.00		above 7.00	

	Mean	Standard Deviation	Mean	Standard Deviation
Age	57.07	8.19	54.45	11.15
DMYRS	7.33	6.03	7.38	5.57
BMI	24.00	2.67	27.26	3.50
CHO	154.11	38.85	183.62	50.79
TGs	138.56	37.93	175.39	61.01
LDL	97.85	27.53	107.59	38.98
HDL	42.26	12.56	39.84	11.98
CHO/HDL	4.64	1.31	4.95	3.01
HbA1c	6.18	0.57	10.23	1.99

The risk of developing chronic complications of diabetes in both genders and shows significant association of metabolic syndrome in female gender. Frequency of chronic complications of diabetes in both genders shows interesting observations as HTN seems to be positive in 50% of male diabetics and 60% of female diabetics as shown in Table-IV Similarly, Metabolic Syndrome was present in 13 % of males (5/39) and 43% of females (44/ 103) which shows a significant gender-based difference.

**Table-IV:** Frequency of chronic complications of diabetes in Male and Female genders (n# 142).

	Males (n#39)		Females(n#103)	

	Yes	No	Yes	No
Hypertension	19	20	61	42
Metabolic Syndrome	5	34	44	59
Ischemic Heart Disease	7	32	14	89
Chronic Kidney Disease	9	30	10	93
Cerebrovascular Accident	1	38	2	101

## DISCUSSION

Elevated HbA1c in addition to dyslipidemia has now been regarded as an independent risk factor for CVD in subjects with or without diabetes. Estimated risk of CVD has shown to be increased by 18% for each 1% increase in absolute HbA1c value in diabetic population. Positive relationship between HbA1c and CVD has been demonstrated in non-diabetic cases even within normal range of HbA1c.^8^

Insulin resistance has a major role in the pathogenesis of diabetic dyslipidemia as there is evidence of increased release of free fatty-acid from insulin resistance fat cells. The free fatty acids entering into the liver in the presence of glycogen promotes triglyceride production and also secreation of apolipoprotein B and VLDL cholesterol, thus resulting in fatty liver. Similarly, higher levels of circulating insulin is also associated with low HDL levels.^9^ As one of the manifestations of insulin resistance is a higher HbA1c therefore, it may be a common factor in the present study, although insulin is not measured in our study to confirm the hypothesis but the BMI and presence of metabolic syndrome supports our hypothesis of insulin resistance associated increasing HbA1c, these factors can result as a consequence in dyslipidemia as already discussed.

Glycated hemoglobin (HbA1c) is a routinely used marker for long-term glycemic control as it is user friendly. In addition, it is a stable test with minimal biological variability and is not affected by factors which otherwise has considerable impact on glucose measurement.^10^

The concentration of HbA1c predicts diabetes complications because it reflects more harmful glycation sequelae of diabetes, such as retinopathy and nephropathy due to harmful advanced glycation end products.^11^

Mean HbA1c levels in our study in both the genders is approximately the same however, the lipid profile is deranged more in the female population, the observation seems very interesting in comparison with the hypothesis that somehow, HbA1c is responsible for the dyslipidemia. Therefore, their seems to be some hidden factors responsible for this gender based difference, one of which is the post menopausal state (mean age in female subjects is 54 yrs ± 11.4 SD) in most of the female population which results in low estrogen production which is considered cardioprotective. This is supplemented by one of the study done by Jacob which endorses that the exposure of estrogen is negatively associated with Cardiovascular events as well as LDL and non HDL cholesterol.^12^

In the present study, BMI, CHO LDL and TG’s have shown a very strong correlation (p< 0.05) with each other as well as with increasing HbA1c whereas inverse relationship is seen between HbA1c and HDL. This observation is proven in many international studies although data from this geographical region is still limited. Naqvi et al has done a similar study in our setting and has some interesting results showing that high HbA1c (cut-off of 9%) increases the risk of hypertriglyceridemia by 2.69 (OR=1.71-4.23, p <0.001) or poor glycemic control can increase the risk of hypertriglyceridemia by 2.69%^13^.

Metabolic Syndrome in our study has shown a very strong association with a higher HbA1c, esp in the female gender (44/103) with a P value of 0.001. A total of 49(34.5%) patients out of 142 had and 93 did not have Metabolic syndrome with a frequency of 42.7 % in females and 7% in males, this observation also endorsed the finding in a study done in Multan which shows 43% females of Type-2 DM has Metabolic Syndrome.^14^ A similar gender difference in prevalence of Metabolic syndrome is observed in a study done by Seerat and Saroj which revealed that 29% of females and 23% of males when screened were having Metabolic Syndrome. They observed that elevated BMI, low HDL cholesterol, increased waist circumference and hyperglycemia proved strong contributors to females causing metabolic syndrome, whereas in males hypertension and elevated triglycerides were the main reasons responsible for this alarming comorbid.^15^

The gender-specific pathophysiological differences in Metabolic Syndrome has also been attributed to prevalence of dysglycemia, fat partitioning, adipocyte biology, hormonal regulation of body weight and adiposity, and effects of estrogen in several studies including the study done by Pradhan.^16^

We have proved in our study that hypertension has the strongest correlation with Type-2 DM as 50% of our male subjects and 60% of female subjects have hypertension in addition to Type-2 diabetes. Our finding is in agreement with the earlier study by Lastra which concluded that 50% of their subjects with type 2 diabetes had hypertension as comorbid.^17^

The hyperinsulinemia, which is the hallmark of insulin resistance in Type-2 diabetes leads to vascular smooth muscle cell proliferation thus increased vascular stiffness, in addition it also impairs vasodilation, increase oxidative stress and the inflammatory process in the vascular wall. The summative effect is the impaired autoregulation of vascular tone, increased vascular resistance, and BP elevation. The antinatriuretic properties of insulin which increases renal retention of sodium also aids in the development of hypertension.^18^

### Limitations and recommendations

In our study sample size is small therefore, further large sample sized RCT’s are required to endorse or refute the findings from our region in different settings. Early intervention for screening all diabetic patients with a high HbA1c to confirm the presence of dyslipidemia and aggressive treatment approach is recommended to lower the cardiovascular morbidity and mortality in Type-2 diabetic patients.

## CONCLUSION

Our study concludes that HbA1c provides valuable supplementary information about the extent of circulating lipids besides its primary role in monitoring long-term glycemic control. Thus, dual biomarker capacity of HbA1c (glycemic control as well as lipid profile indicator) may be utilized for screening high-risk diabetic patients belonging to lower socioeconomic strata. As elevated HbA1c and dyslipidemia are independent risk factors of Cardiovascular disease, diabetic patients with elevated HbA1c and dyslipidemia can be considered as a very high risk group for a ischemic event. Improving glycemic control by an aggressive interventional approach and timely intervention with statins can substantially reduce or delay the risk of those subjects especially who has a higher HbA1c with dyslipidemia but hasn’t developed ischemic heart disease yet.

### Authors’ Contribution:

**SSK:** Conceived the idea, designed the study, compiled the results & discussed the observations. Also, author for correspondence who is accountable for the accuracy or integrity of the work.

**AN:** Collected and analysed the data.

**FB:** Write-up and critical analyses of manuscript.

**JA:** Concept of study, manuscript writing and final review.

## References

[1] Sherwani IS, Khan AH, Ekhzaimy A, Masood A, Sakharkar MK (2016). Significance of HbA1c Test in Diagnosis and Prognosis of Diabetic Patients. Biomarker Insights.

[2] Leon MB, Maddox MT (2015). Diabetes and cardiovascular disease: Epidemiology, biological mechanisms, treatment recommendations and future research. World J Diabetes.

[3] Alam R, Verma KM, Verma P (2015). Glycated Hemoglobin as a Dual Biomarker in Type 2 Diabetes Mellitus Predicting Glycemic Control and Dyslipidemia Risk. Int J Life-Sci Scient Res.

[4] Sarfraz M, Sajid S, Ashraf MA (2016). Prevalence and pattern of dyslipidemia in hyperglycemic patients and its associated factors among Pakistani population.

[5] Verges B (2009). Lipid modification in type 2 diabetes:the role of LDL and HDL. Fundam Clin Pharmacol.

[6] Nathan MD (2014). The Diabetes Control and Complications Trial/Epidemiology of Diabetes Interventions and Complications Study at 30 Years. Diabetes Care.

[7] (2018). AHA- Guideline on the Management of Blood Cholesterol:Executive Summary. A Report of the American College of Cardiology/American Heart Association Task Force on Clinical Practice Guidelines. National Guidelines:10 Nov.

[8] Hussain A, Ali I, Ijaz M, Rahim A (2017). Correlation between hemoglobin A1c and serum lipid profile in Afghani patients with type 2 diabetes:hemoglobin A1c prognosticates dyslipidemia. Ther Adv Endocrinol Metab.

[9] Momin AA, Bankar MP, Bhoite GM (2013). Glycosylated hemoglobin (HbA1C):Association with dyslipidemia and predictor of cardiovascular diseases in type 2 diabetes mellitus patients. Int J Health Sci Res.

[10] Use of Glycated Haemoglobin (HbA1c) in the Diagnosis of Diabetes Mellitus:Abbreviated Report of a WHO Consultation (2011). Glycated haemoglobin (HbA1c) for the diagnosis of diabetes.

[11] Ketema EB, Kibret KT (2015). Correlation of fasting and postprandial plasma glucose with HbA1c in assessing glycemic control;systematic review and meta-analysis. Arch Public Health.

[12] Christ JP, Gunning MN, Palla G, Eijkemans MJC, Lambalk CB, Laven JSE (2018). Estrogen deprivation and cardiovascular disease risk in primary ovarian insufficiency. Fertil Steril.

[13] Naqvi S, Naveed S, Ali Z, Ahmad MS, Khan AR (2017). Correlation between Glycated Hemoglobin and Triglyceride Level in Type 2 Diabetes Mellitus. Cureus.

[14] Tanweer S, Illahi Y, Amatya B, Naeem A, Tareen ZF (2011). Frequency of the metabolic syndrome in type 2 diabetic subjects attending the diabetes clinic of Nishtar Medical College and Hospital, Multan. Ann Punjab Med Coll.

[15] Beigh HS, Jain S (2012). Prevalence of metabolic syndrome and gender differences. Bioinformation.

[16] Pradhan DA (2014). Sex Differences in the Metabolic Syndrome:Implications for Cardiovascular Health in Women. Clin Chem.

[17] Lastra G, Syed S, Kurukulasuriya LR, Manrique C, Sowrs JR (2014). Type 2 diabetes mellitus and hypertension:an update. Endocrinol Metab Clin North Am.

[18] Villalpando GC, Meigs BJ, Ferrannini E (2018). Hypertension and Diabetes Mellitus. Coprediction and Time Trajectories. Hypertension.

